# Supplementary figures and images for: The UBR-1 ubiquitin ligase regulates glutamate metabolism to generate coordinated motor pattern in Caenorhabditis elegans
Source: PLoS Genet. 2018 Apr 12;14(4):e1007303. doi: 10.1371/journal.pgen.1007303 (PMC5931689; doi:10.1371/journal.pgen.1007303)

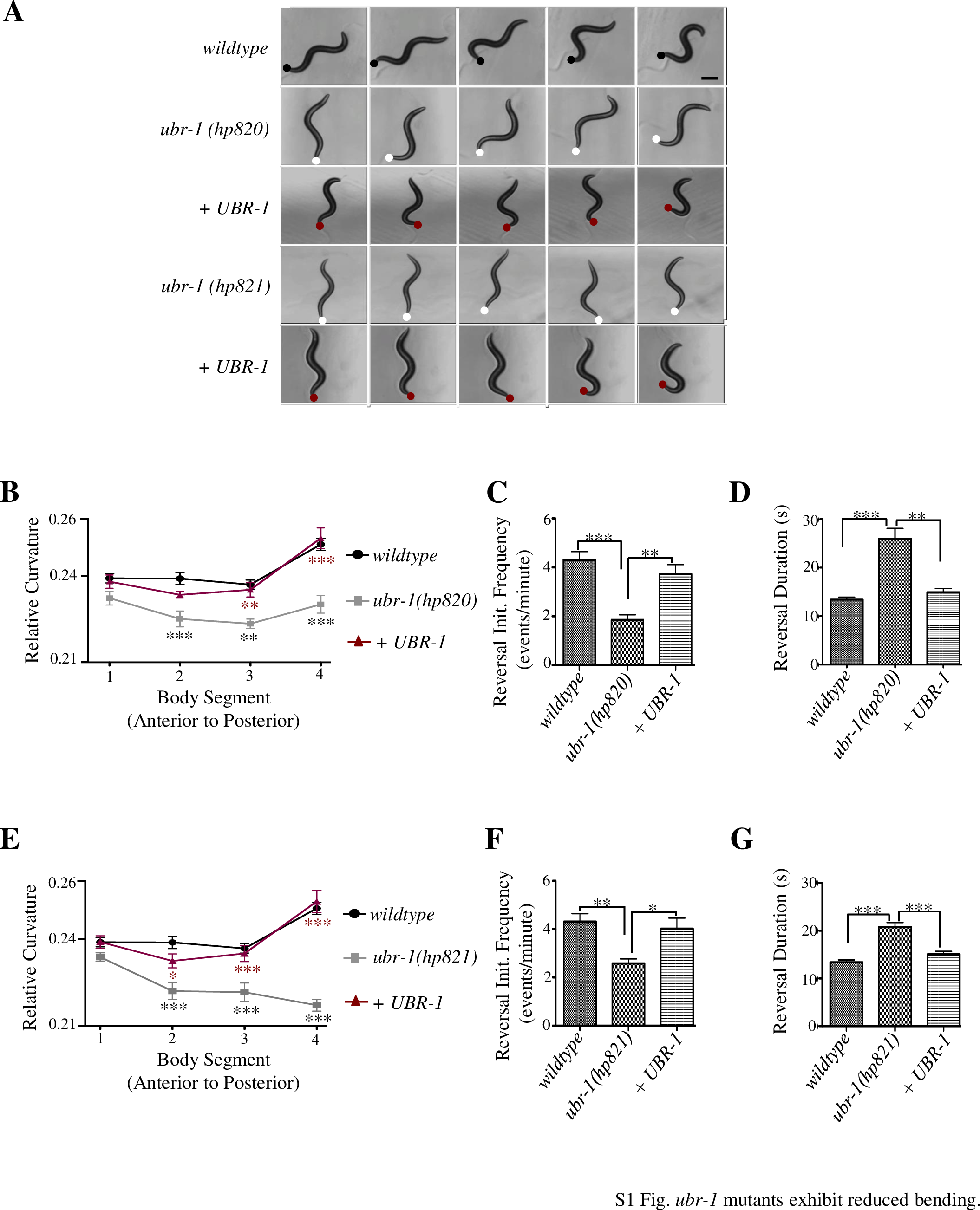

Supplement: S1 Fig — A) Representative images of wildtype animals and two alleles of ubr-1 mutants during reversals (left to right panels). The wildtype animal generates sinusoidal body bends, whereas ubr-1(hp820, hp821) animals do not bend. This defect was rescued by restoring the expression of UBR-1. Dots denote position of tail. Scale bar: 200μm. In ubr-1(hp820) (B) and ubr-1(hp821) (E) alleles (grey line), bending curvature is reduced throughout head to tail compared to wild type (black line), and this was rescued by restoring expression of UBR-1 using its endogenous promoter (red line). ubr-1 mutants have fewer initiations (C, F) and longer durations for reversals (D, G). ***P<0.001, **P<0.01, *P< 0.05 by the Two-way RM ANOVA test. Data are represented as mean ± SEM. ***P<0.001, **P<0.01, *P<0.05 by the Kruskal-Wallis test. Data are represented as mean ± SEM. (TIF) [file pgen.1007303.s001.tif]

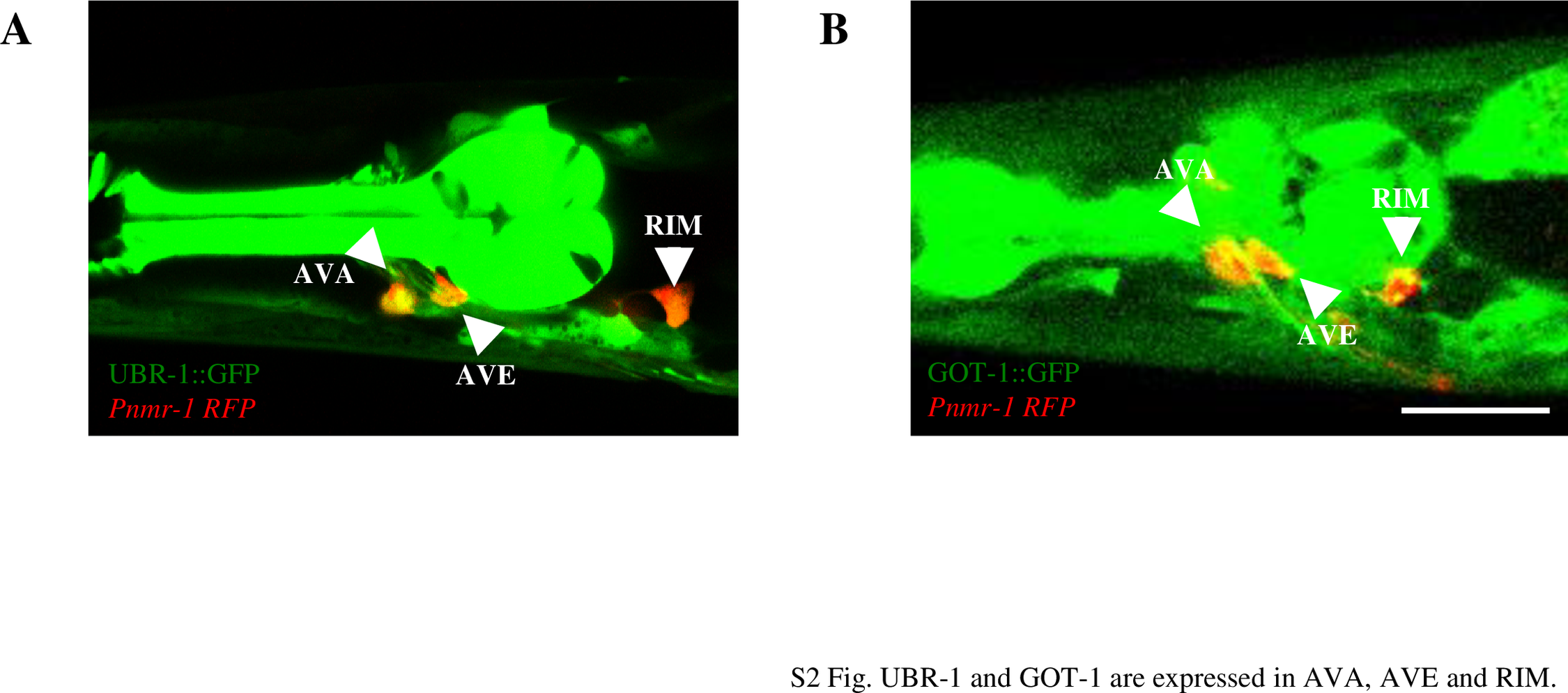

Supplement: S2 Fig — A) A confocal image of animals expressing a functional UBR-1::GFP transgene from its endogenous promoter (green), and the Pnmr-1-RFP reporter (red). B) A confocal image of animals expressing endogenous GOT-1::GFP from the knock-in allele (green), and the Pnmr-1-RFP reporter (red). Robust expression of UBR-1 and GOT-1 was present at the pharynx; neuronal UBR-1::GFP and GOT-1::GFP signals were present in the AVA, AVE and RIM premotor interneurons (denoted), and other unidentified neurons (not shown). Scale bar, 5 μm. (TIF) [file pgen.1007303.s002.tif]

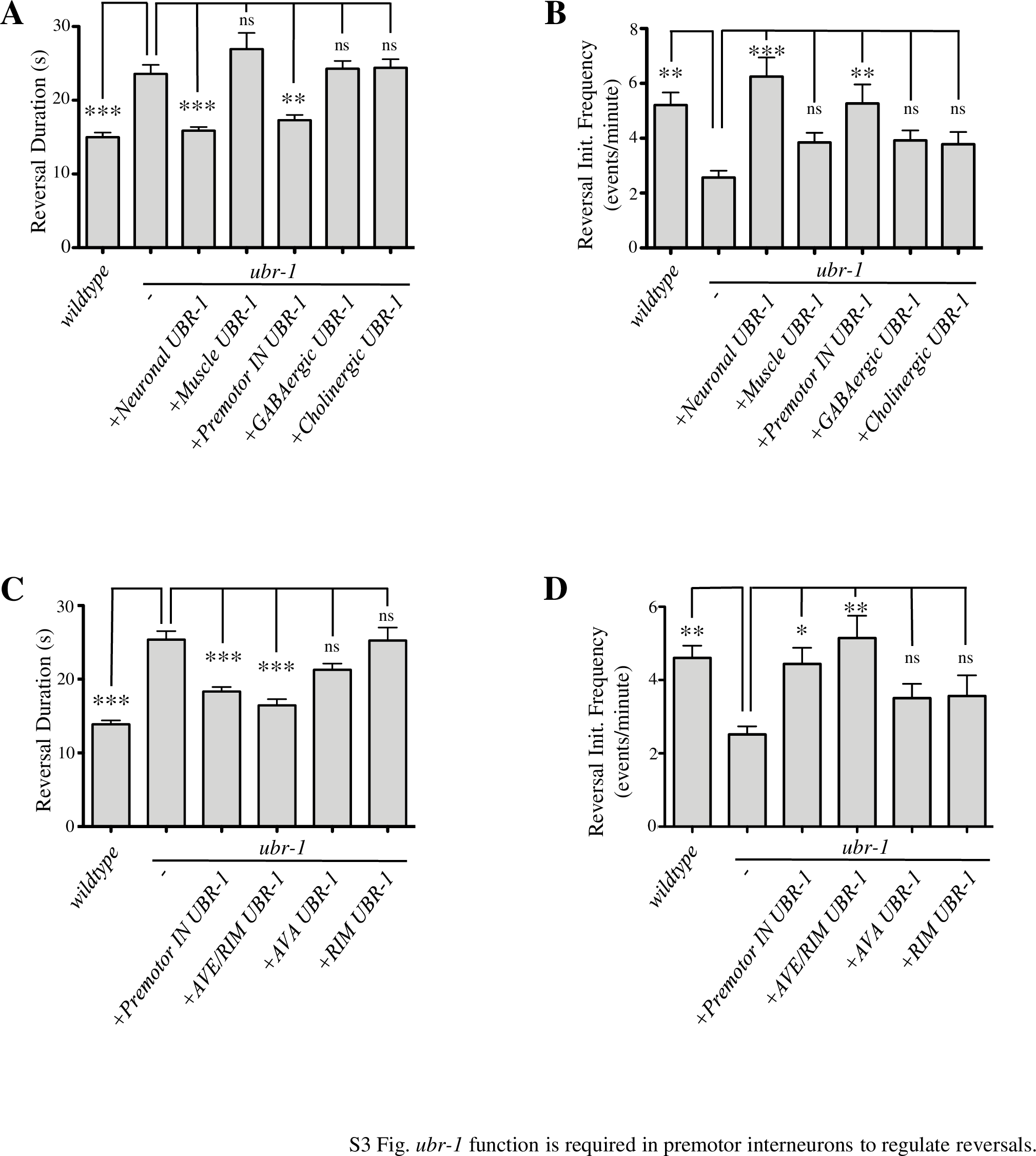

Supplement: S3 Fig — A-B) In ubr-1 mutants, the reversal duration is increased, while reversal initiation frequency is decreased. Both the parameters were rescued by restoring the expression of UBR-1 in premotor interneurons, but not in GABAergic or cholinergic motor neurons. C-D) Expression of UBR-1 in multiple premotor interneurons, which include AVE/RIM exhibited significant rescue of ubr-1 for reversal duration and initiation frequency. Expression of UBR-1 in the RIM alone or in AVA alone did not result in rescue. *P<0.05, **P<0.01, ***P<0.001 by the Kruskal-Wallis test. Data are represented as mean ± SEM. (TIF) [file pgen.1007303.s003.tif]

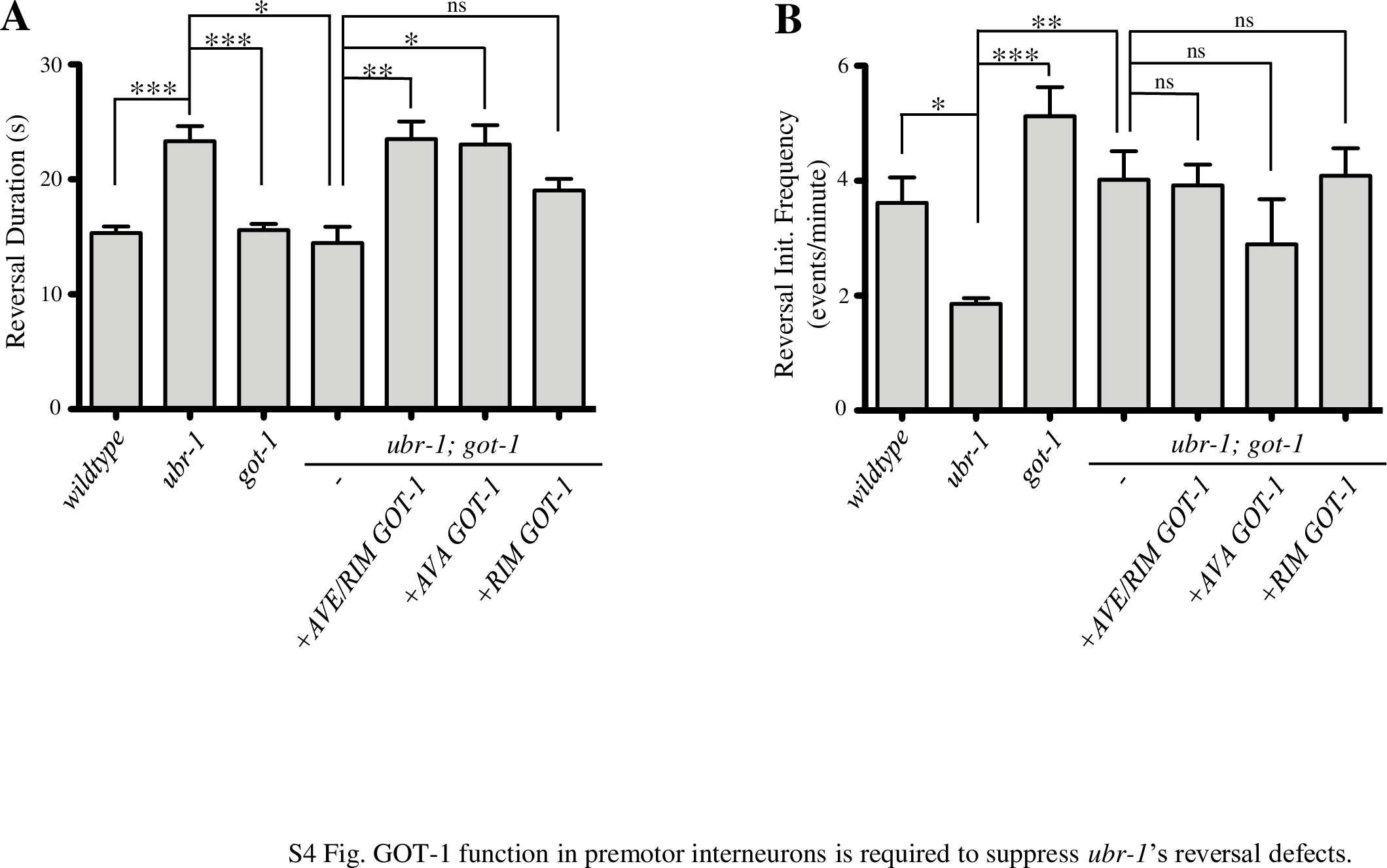

Supplement: S4 Fig — The functional loss of got-1 restores the reversal duration (A) and the reversal initiation frequency (B) in ubr-1 mutants. Restoring GOT-1 expression in multiple premotor interneurons, including AVE and RIM, reverted the reversal duration of ubr-1; got-1 to that of ubr-1 (A), whereas the reversal initiation frequency exhibited the trend of decrease but did not reach statistic significance (B). Expression of GOT-1 in RIM alone had no effect, while the expression of GOT-1 in AVA partially reverted the reversal duration. *P<0.05, **P<0.01, ***P<0.001 by the Kruskal-Wallis test. Data are represented as mean ± SEM. (TIF) [file pgen.1007303.s004.tif]

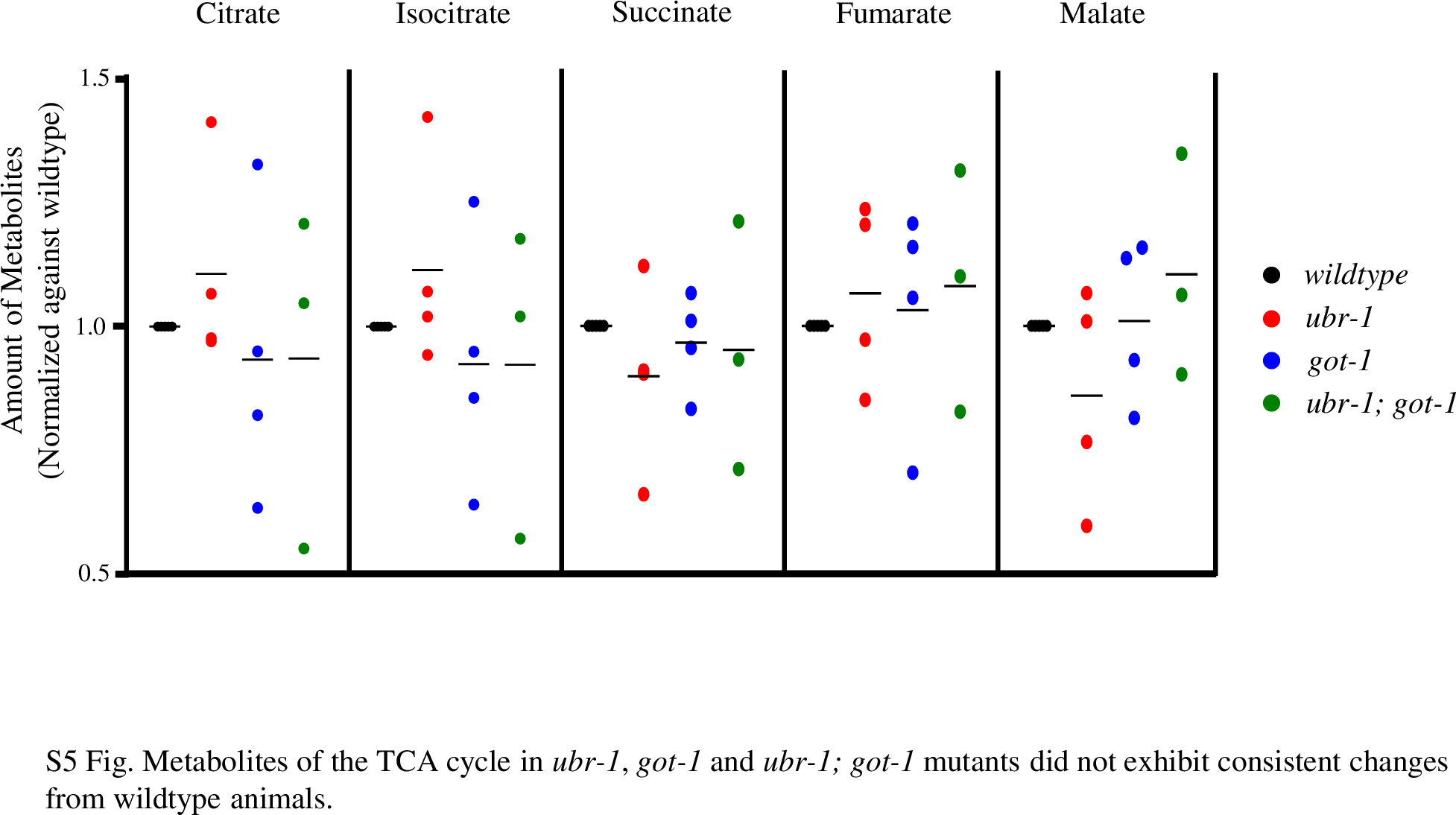

Supplement: S5 Fig — Metabolite levels measured by LC-MS, normalized against the total protein level in the lysate. All mutants were normalized to that of wildtype animals. Metabolites of the TCA cycle did not show any coordinated changes among these mutants. (TIF) [file pgen.1007303.s005.tif]

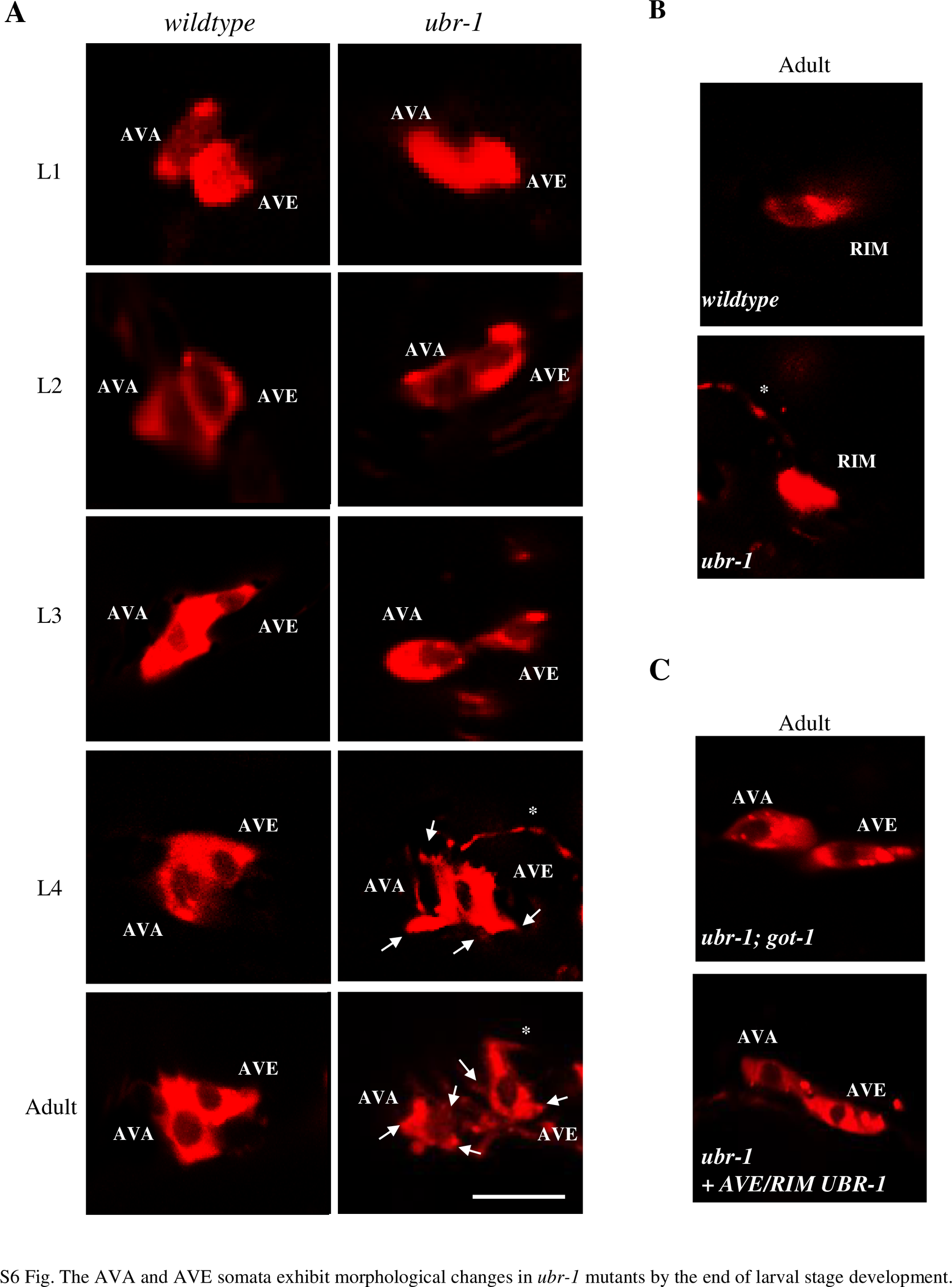

Supplement: S6 Fig — A) Confocal images of the AVA and AVE premotor interneuron somata in wildtype and ubr-1 animals, which were labeled with cytosolic RFP, from the L1 juvenile larva to adult stages. Depending on the focal planes, some images contain that of nuclei, which were devoid of RFP signals. Left panels: images from wildtype animals. The surface of both somata was round and smooth throughout larval development and in young adults. Right panels: images from ubr-1 mutants. In young (L1 to L3) larva, somata appeared similar to those in age-matched wildtype animals. In the L4 larva and adult animals, somata developed rough surface and short branches, denoted by arrows. B) The RIM soma exhibited normal morphology in adults, similar to wildtype animals. C) The round morphology AVA and AVE somata in ubr-1 adults were restored in ubr-1; got-1 double mutant adults, and in ubr-1 adults with restored UBR-1 expression in multiple premotor interneurons including AVE and RIM. Asterisks (*) mark the AVA or AVE axons, which could not be shown in some panels when they were in a different focal plane as the somata. Scale bar, 2μm (for the L1 and L2 panels) 5 μm (for the L3 to adult panels). (TIF) [file pgen.1007303.s006.tif]
