# Supplementary material for: The UBR-1 ubiquitin ligase regulates glutamate metabolism to generate coordinated motor pattern in Caenorhabditis elegans
Source: PLoS Genet. 2018 Apr 12;14(4):e1007303. doi: 10.1371/journal.pgen.1007303 (PMC5931689; doi:10.1371/journal.pgen.1007303)
Supplement: S1 Table — (DOCX) [file pgen.1007303.s010.docx]

**Supporting Information**

**Table S1. Strains generated or acquired for this study.**

a. Non-transgenic strains generated for genetic interactions with *ubr-1*

| **Strain Names** | **Genotypes** |
| --- | --- |
| ZM8230 | *ubr-1(hp684)* |
| ZM8219 | *ubr-1(hp820)* |
| ZM8201 | *ubr-1(hp821)* |
| ZM8202 | *ubr-1(hp821 hp833)* |
| ZM6660 | *got-1 (hp731)* |
| ZM8226 | *ubr-1(hp684); got-1 (hp731)* |
| ZM9104 | *ubr-1 (hp684); got-1(hp731)/mnDf1* |
| ZM7589 | *ubr-1 (hp684); got-1.1 (tm2311)* |
| ZM7580 | *ubr-1 (hp684); got-1.3 (tm3424)* |
| ZM8394 | *ubr-1(hp684) got-2.1 (gk109644 )* |
| ZM7373 | *ubr-1 (hp684); unc-25 (e156)* |
| ZM7903 | *ubr-1(hp684); gln-1 (gk329791)* |
| ZM7904 | *ubr-1(hp684); glna-2 (gk536170)* |
| ZM7677 | *ubr-1(hp684); C32F10.8 (tm2997)* |
| ZM7588 | *ubr-1(hp684); gta-1 (ok517)* |
| ZM7905 | *ubr-1(hp684); snf-11 (ok156)* |
| ZM7291 | *ubr-1(hp684); idhb-1 (ok2368)* |
| ZM7583 | *ubr-1(hp684); pdhk-2 (tm3233)* |
| ZM7584 | *ubr-1(hp684); pyc-1 (tm3233)* |
| ZM7908 | *ubr-1(hp684); pyk-1 (ok1754)* |
| ZM7585 | *ubr-1(hp684); T28F3.5 (tm4283)* |
| ZM7591 | *ubr-1(hp684); rsks-1 (ok1255)* |
| ZM10005 | *ubr-1(hp865) lin-28(n719)* |
| ZM9971 | *ubr-1(hp865) lin-28(n947)* |
| ZM8368 | *ubr-1(hp684);ced-3(n717) hpIs202* (* RID marker)* |
| ZM8368 | *ubr-1(hp684);ced-4 (n1162) hpIs316* (* RID marker)* |

b. Transgenic strains generated for A-MN calcium imaging

| **Strain**  **Names** | **Genetic**  **Backgrounds** | **Transgenes** | **Plasmid Names**  **(Description)** |
| --- | --- | --- | --- |
| ZM8426 | *-* | *hpIs460* | pJH3137 (*Punc-4-GCaMP6s::wCherry)* |
| ZM8749 | *ubr-1 (hp684)* |  |  |
| ZM8750 | *ubr-1 (hp684); got-1 (hp731)* |  |  |
| ZM8846 | *ubr-1(hp684)* | *hpIs460* | pJH3137 (*Punc-4-GCaMP6s::wCherry)* |
|  |  | *hpEx3676* | pJH3476 (*Popt-3::UBR-1 minigene)* |

**c. Transgenic strains generated for behavioral rescue, gene expression, and biochemistry experiments.**

| **Gene** | **Strain** | **Backgrounds** | **Trans-genes** | **Plasmids** |
| --- | --- | --- | --- | --- |
| **U**  **B**  **R** | ZM7440 | *ubr-1(hp684)* | *hpEx3161* | pJH2913 *(Pubr-1::UBR-1)* |
|  | ZM8272 | *ubr-1(hp821hp833)* | *hpEx3482* |  |
|  | ZM8270 | *ubr-1(hp820)* | *hpEx3480* |  |
|  | ZM8271 | *ubr-1(hp821)* | *hpEx3481* |  |
|  | ZM7410 | *lin-15(n765)* | *hpEx3056* | pJH2963 (*Pubr-1::GFP)* |
|  | ZM8628 | *-* | *hpIs487* | pJH2965 (*Pubr-1::GFP::UBR-1)* |
|  | ZM8389 | *ubr-1(hp684)* | *hpEx3535* | pJH3380 (*Prgef-1::UBR-1 minigene)* |
|  | ZM8388 |  | *hpEx3536* | pJH3381 (*Pmyo-3::UBR-1 minigene)* |
|  | ZM8390 |  | *hpEx3537* | pJH3382 (*Punc-47::UBR-1 minigene)* |
|  | ZM8391 |  | *hpEx3538* | pJH3389 (*Pacr-2::UBR-1 minigene)* |
|  | ZM8404 |  | *hpEx3541* | pJH3390 (*Pglr-1::UBR-1 minigene)* |
|  | ZM8457 |  | *hpEx3564* | pJH3436 (*Pnmr-1::UBR-1 minigene)* |
|  | ZM8551 |  | *hpEx3595* | pJH3469 (*Prig-3::UBR-1 minigene)* |
|  | ZM8550 |  | *hpEx3596* | pJH3476 (*Popt-3::UBR-1 minigene)* |
|  | ZM8582 |  | *hpEx3597* | pJH3485 (*Plgc-55s::UBR-1 minigene)* |
|  | ZM8627 |  | *hpEx3622* | PJH3502 (*Pgcy-13::UBR-1 minigene)* |
|  | ZM8580 |  | *hpEx3599* | PJH3487 (*Pttx-3::UBR-1 minigene)* |
|  | ZM8581 |  | *hpEx3598* | PJH3486 (*Pinx-1::UBR-1 minigene)* |
| **G**  **O**  **T** | ZM7289 | *TTti5605* | *hpSi10* | pJH2998 (*GOT-1.2::GFP MosCI)* |
|  | ZM8458 |  | *hpSi16* | pJH3396  (*Prgef-1::GOT-1.2 MosCI)* |
|  | ZM8460 |  | *hpSi18* | pJH3397  (*Pglr-1::GOT-1.2 MosCI)* |
|  | ZM8462 |  | *hpSi20* | pJH3399  (*Punc-47::GOT-1.2 MosCI)* |
|  | ZM8497 |  | *hpSi22* | pJH3471 (*Prig-3::GOT-1.2 MosCI)* |
|  | ZM8760 | *ubr-1(hp684);*  *got-1(hp731)* | *hpEx3661* | pJH3531 (*Popt-3::GOT-1.2)* |
|  | ZM8759 |  | *hpEx3621* | pJH3501 (*Pgcy-13::GOT-1.2)* |
|  | ZM8102 |  | *hpEx3998* | pJH2896 (*Pmyo-3::GOT-1.2)* |
|  | ZM8631 | *-* | *hpIs484* | pJH3437 (*Prgef-1::GOT-1::GFP)* |
|  | ZM9008 | *-* | *hp881* | pJH3621 *(targeting construct for generating C-terminal GFP insertion at the endogenous locus of GOT-1.2)* |
|  | ZM9077 | *ubr-1(hp684)* |  |  |
|  | ZM9084 | *ubr-1 (hp821hp833)* |  |  |
